# Construction of a rAAV-SaCas9 system expressing eGFP and its application to improve muscle mass

**DOI:** 10.1007/s10529-021-03183-1

**Published:** 2021-09-29

**Authors:** Shaoting Weng, Yitian Zhao, Changhong Yu, Xiaofan Wang, Xuehan Xiao, Liqiang Han, Kunpeng Zhang, Jiang Wang, Guoyu Yang

**Affiliations:** 1 Department of Animal Biotechnology, Henan Agricultural University, Zhengzhou, 450002 Henan People’s Republic of China; 2 Department of Biotechnology, Anyang Institute of Technology, Anyang, 455000 Henan People’s Republic of China

**Keywords:** Genome editing, rAAV-SaCas9 system, eGFP protein, Myostatin, Skeletal muscle proliferation

## Abstract

An ideal rAAV gene editing system not only effectively edits genes at specific site, but also prevents the spread of the virus from occurring off-target or carcinogenic risks. This is important for gene editing research at specific site in vivo. We report a single rAAV containing SaCas9 and guide RNAs under the control of subtle EF1a and tRNA promoters. The capacity of rAAV was compressed, and the editing efficiency was similar to that of the classical Cas9 system in vitro and in vivo. And we inserted the sequence of the green fluorescent protein eGFP into rAAV. The number of cells infected with the rAAV and the region in which the rAAV spreads were known by the fluorescent expression of eGFP in cells. In addition, we demonstrated that myostatin gene in the thigh muscles of C57BL/10 mice was knocked out by the rAAV9-SaCas9 system to make muscle mass increased obviously. The protein eGFP into rAAV has significant implications for our indirect analysis of the editing efficiency of SaCas9 in the genome of the target tissue and reduces the harm caused by off-target editing and prevents other tissue mutations. The rAAV system has substantial potential in improving muscle mass and preventing muscle atrophy.

## Introduction

The CRISPR/Cas9 system has become a commonly used technology in medicine and life sciences (Li et al. 2018). It has been used to genetically modify potential clinical treatments for diseases (Mollanoori and Teimourian 2018; Yang et al. 2016). However, genome editing in tissues in vivo is affected by many factors, including the choice of vector, the efficiency of editing proteins and the influence of the internal environment. In clinical settings, adeno-associated virus (AAV) as the vector for Cas9 provides a promising genomic correction approach (Moreno et al. 2018). In particular, the technique allows genetic editing of specific muscle areas of inherited muscular dystrophy to improve atrophy symptoms (Crudele and Chamberlain 2019; Tabebordbar et al. 2016). However, many of the exciting tools developed from CRISPR/Cas9 technology are too bulky to meet the genome packaging limits of AAV (~ 4.85 kb including both ITRs). Notably, SpCas9 is ~ 4.3 kb, and it scarcely fits into the AAV capacity when coupled with essential gene regulatory elements. One way to overcome this technical hurdle is to take advantage of smaller orthologs of Cas9 derived from different prokaryotic species. Streptococcus thermophilus Cas9 (St1Cas9), Neisseria meningitidis Cas9 (NmCas9), and Staphylococcus aureus Cas9 (SaCas9) nucleases that have been recognized to be effective have similar properties to those of Streptococcus pyogenes Cas9 (SpCas9), but are all ~ 1 kb shorter than SpCas9 (Kleinstiver et al. 2015; Esvelt et al. 2013; Ran et al. 2015). Thus, with the discovery of these smaller Cas9 nucleases, AAVs can be engineered with either of these smaller Cas9 genes or gRNA expression cassettes to create AAV-based CRISPR/Cas9. This technical hurdle can also be overcome by optimizing the promoter. Mefferd and coworkers have recently reported that expression of sgRNAs can be driven by small tRNA promoters (~ 70 bp) with sizes approximately half that of an sgRNA-expressing cassette with a U6 promoter (Mefferd et al. 2015) for efficient delivery of CRISPR materials into cells. Tabebordbar and coworkers have reconstructed a new vector AAV-SaCas9 in which expression of SaCas9 is driven by EF1a-short promoters (Tabebordbar et al. 2016). Other researchers have built the pX601-miniCMV-SaCas9-U6-sgRNA vector, in which expression of SaCas9 is driven by a miniCMV promoter, which is only 39 bp. The smaller sizes of two or more components of a single rAAV system would be necessary to perform genome-editing experiments, including rAAV tissue tropism, and efficient precise targeting and persistent performance of organizations (Zincarelli et al. 2008).

Myostatin (*Mstn*, also known as growth differentiation factor 8), is a member of the transforming growth factor β (TGF-b) signaling protein superfamily and a key regulator of muscle mass in vertebrates. Its expression inhibits the proliferation and differentiation of muscle cells. Myostatin signaling dysfunction promotes muscle growth, and myostatin-null animals have a characteristic supermuscle phenotype (McPherron et al. 1997; McPherron and Lee 1997). Unsurprisingly, in muscular dystrophy diseases, including sarcopenia, muscular dystrophy, and cancer-related cachexia, controlling myostatin signaling has become an attractive prospect for increasing functional muscle mass (Camporez et al. 2016; Smith and Lin 2013; Wei et al. 2016; Gallot et al. 2014; Weng et al. 2020). Researchers are increasingly exploring treatments for muscular dystrophy by knocking out *Mstn* gene expression, which has been found to be a simple and rapid way to improve muscle traits (Wei et al. 2016; Rodriguez et al. 2011; Salzler et al. 2016).

Here, we built a smaller rAAV-SaCas9 gene editing system, which generated EF1α-driven SaCas9 and tRNA-driven sgRNAs. And it indirectly understood the editing effect and distribution of Cas9 through the expression of eGFP in vivo and in vitro. Furthermore, we found that knockout of *Mstn* gene in mice muscle by this system effectively increased the mass of muscle cells. This study provides a reference for improving muscle mass and preventing muscle atrophy.

## Materials and methods

### Construction of SaCas9 and sgRNA plasmids

**Fig. 1:**
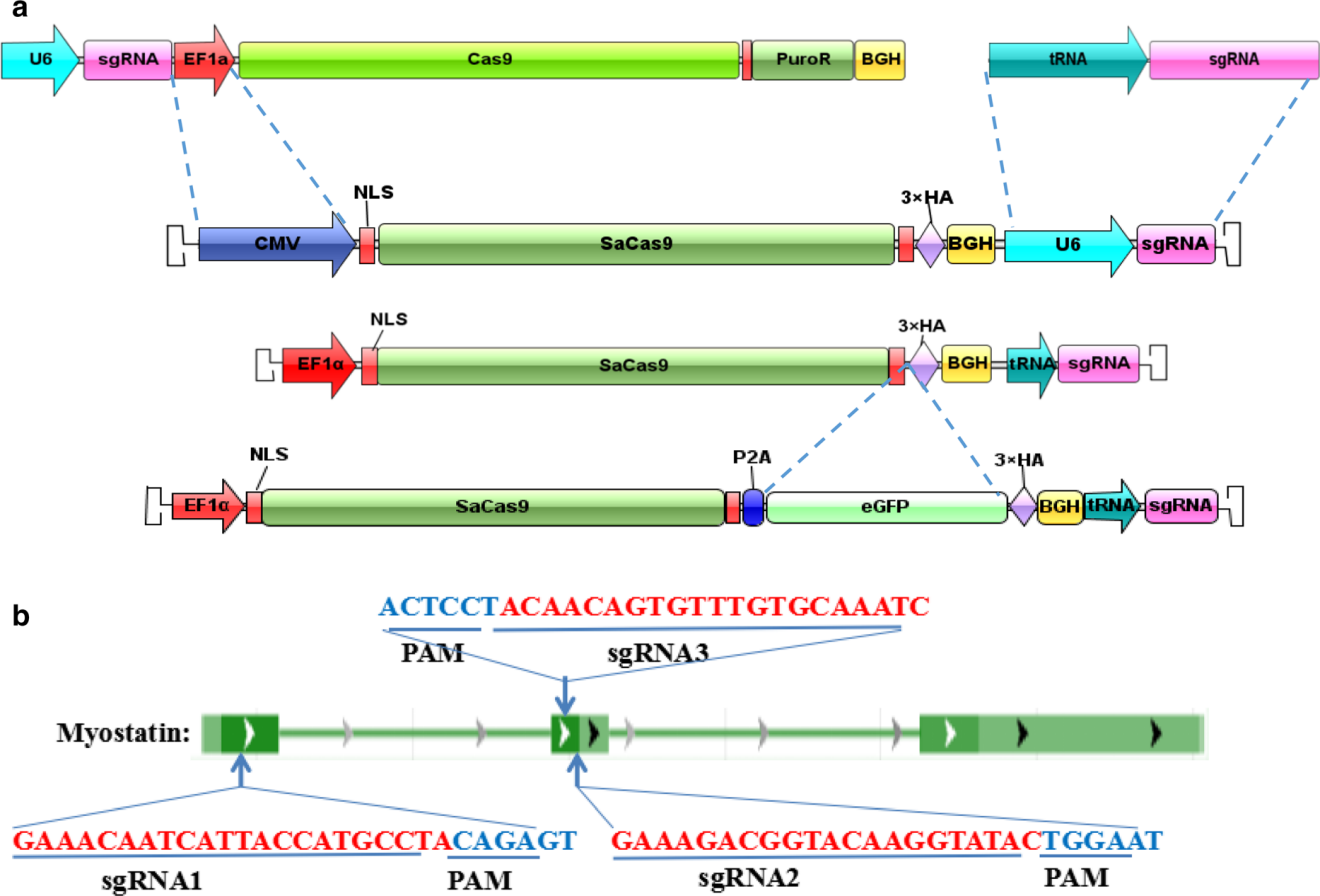
The SaCas9/CRISPR system map depicting EF1α:SaCas9-eGFP-tRNA:sgRNA. **a** EF1α:SaCas9-eGFP-tRNA:sgRNA is an SaCas9/CRISPR system that consists of an EF1α promoter controlling the expression of the coding regions of SaCas9, followed by a self-cleaving P2A peptide sequence and then an eGFP Flag. The sgRNA expression cassette is under the control of an tRNA promoter. **b** The approximate location of the sgRNA binding site in the *Mstn* locus. These three target sites were all located in the conserved N-terminal sequence in the first and second exons of the *Mstn* gene, which play an important role in MSTN protein synthesis. The sgRNAs, or target sequences are underlined, and the PAM sequence (NNGRRT) is specified

All plasmids were constructed with standard recombinant DNA cloning techniques. pX601-CMV:SaCas9-U6:sgRNA (pX601-AAV-CMV::NLS-SaCas9-NLS-U6::*Bsa*I-sgRNA) was preserved in our laboratory. tRNA_GLN_ and sgRNA scaffold sequences were generated by Sangon (Shanghai, China) (GGTTCCATGGTGTAATGGTTAGCACTCTGGACTCTGAATCCAGCGATCCGAGTTCAAATCTCGGTGGAACCT-GAAACACCGGAGACCACGGCAGGTCTCAGTTTTAGTACTCTGGAAACAGAATCTACTAAAACAAGGCAAAATGCCGTGTTTATCTCGTCAACTTGTTGGCGAGA) (Mefferd et al. 2015) and, after amplification with the primers tRNA_GLN_-F: 5′-AGGCATGCTGGGGAGGTACCGGTTCCATGGTGTAATGGTT-3′, Scaf-ITR-R:5′-CTAGGGGTTCCTGCGGCCGCAAAAATCTCGCCAACAAGTTG-3′, were ligated into pX601-CMV:SaCas9-U6:sgRNA, which was digested with *Kpn* I and *Not* I, with a ClonExpress® II One Step Cloning kit (Vazyme Biotech, China). The constructed vector was then transformed into DH5α competent cells, and transformants containing pX601-CMV:SaCas9-tRNA:sgRNA were identified by sequencing. After subculturing, DNA was extracted. The EF1α promoter was amplified from the pLentiCRISPR V2 vector with the primers EF1α-F: 5′-CCTGCGGCCTCTAGACTCGAGGTGGGCAGAGCGCACATCGC-3′ and EF1α-R: 5-TGGGGCCATGGTGGCACCGGTCCTGTGTTCTGGCGGCAAAC-3′. Then, pX601-CMV:SaCas9-tRNA:sgRNA, which was digested between the *Xho* I and *Age* I sites immediately before the SaCas9 gene, and the promoter were cloned with a ClonExpress® II One Step Cloning kit. Vector transformation, identification and plasmid extraction were performed as described above to obtain pX601-EF1α:SaCas9-tRNA:sgRNA. The P2A-eGFP gene was amplified from th-P2A-eGFP donor with primers P2A-eGFP-F: 5′-CCGGCCAGGCAAAAAAGAAAAAGGCTACTAATTTCTCCT-3′ and P2A-eGFP-R: 5′-ATCTGGAACATCGTATGGGTCTTGTACAGCTCGTC. Then, pX601-EF1α:SaCas9-tRNA:sgRNA was digested with *BamH*I, and the P2A-eGFP gene was cloned with a ClonExpress® II One Step Cloning kit. Vector transformation, identification and plasmid extraction were performed as described above to obtain pX601-EF1α:SaCas9-eGFP-tRNA:sgRNA. The proposed structures of the vector are shown in Fig. 1a. The complementary oligonucleotides sgMstn1, sgMstn2 and sgMstn3 were annealed to form double-stranded inserts and were ligated into the *Bbs*I digested plasmid. Finally, the plasmids pX601-EF1α:SaCas9-eGFP-tRNA:sgMstn1, pX601-EF1α:SaCas9-eGFP-tRNA:sgMstn2 and pX601-EF1α:SaCas9-eGFP-tRNA:sgMstn3 (denoted psgMstn1, psgMstn2 and psgMstn3) were constructed.

### Cell culture and transfection

NIH/3T3 cells were cultured in Dulbecco’s modified Eagle’s medium (DMEM) supplemented with 10% fetal bovine serum (FBS) and were transfected with polyethylenimine (PEI). Briefly, 5 × 10^5^ NIH/3T3 cells were cultured to 50–60% confluence in six-well plates and transfected with psgMstn1/psgMstn2/psgMstn3 and 1 × PEI, and the medium was replaced with DMEM (3% FBS) after 8 h. And the control cells were wild type cells cultured in the same batch. After 72 h, the cells were subjected to genomic DNA extraction. All cells were cultured at 37 °C and 5% CO_2_.

### AAV vectors for the CRISPR/Cas9 system

Procedures were carried out as previously described (Senis et al. 2014). For the production of rAAV-DJ/8, rAAV9 viruses, HEK293T cells were seeded into 20 dishes (100 × 10 mm; 5 × 10^6^ cells per dish). After 24 h, the cells were triple-transfected with psgMstn1, pAAV-DJ/8-RC/pAAV9-RC and pHelper according to the instructions provided with the PEI transfection reagent. After 72 h, the cells were harvested by scraping into medium, centrifuged at 1000×*g* for 10 min and resuspended in 1 mL of 1 × phosphate-buffered saline (PBS). The cell suspension was subjected to three freeze–thaw cycles at − 80 °C and at 37 °C. After fast centrifugation and filtration, the cell debris was cleared. The viral solution was concentrated with PEG 8000 and purified on a cesium chloride density gradient column. After two rounds of ultracentrifugation, the high-density viruses were separated and extracted, and run through dialysis bags for desalting (Huang et al. 2017). The titers of the purified rAAV-DJ/8, rAAV9 viruses were determined with a RT-PCR-based method described previously (Holehonnur et al. 2014). pX601 was diluted from 10^9^ copies/μL to 10^3^ copies/μL as the standard solution. The primers were ITR-QPCR-F: 5′-CGGCCTCAGTGAGCGA-3′ and ITR-QPCR-R: 5′-AGGAACCCCTAGTGATG-3′. The resulting viruses were designated AAV-DJ/8-EF1α:SaCas9-eGFP-tRNA:sgMstn1, AAV9-EF1α:SaCas9-eGFP-tRNA: sgMstn1 (denoted rAAV-DJ/8-sgMstn1, rAAV9-sgMstn1, respectively).

### rAAV-DJ/8 transduction in vitro

AAV serotypes differ broadly in transduction efficacy and tissue tropism. Recombinant AAV-DJ vectors provide superior in vitro transduction efficacy to that of any other wild type serotype. Efficient transduction is beneficial for gene editing in the SaCas9 system. After digestion and resuspension of C2C12 cells in logarithmic growth phase, the cells were cultured in DMEM supplemented with 10% FBS in 12-well plates at a seeding density of 1 × 10^5^ and grown overnight. The cells were cultured to 50–60% confluence and infected with ~ 2 × 10^9^ vg/1 × 10^10^ vg rAAV-DJ/8-sgMstn1, and the control cells were wild type cells cultured in the same batch. Then, the medium was replaced with DMEM (3% FBS) after 8 h. Fluorescence was determined, and DNA and protein were extracted at 96 h, 120 h.

### Mice

90 C57BL/10 male mice in SPF grade (6 weeks of age) were purchased from the Center of Experimental Animal of Guangdong province (Guangzhou, China) and maintained in a specific-pathogen-free animal facility according to the Guide for the Care and use of Laboratory Animals and the related ethical regulations at Henan Agricultural University. Then, these mice were fed to the age of 8 weeks and were healthy without any abnormalities.

### rAAV9 transduction in vivo

Compared with AAV-DJ, AAV9 is more addicted in muscle tissue; it is conducive to gene editing of muscle cells and is often applied in muscle tissue. In addition, the packaging of different types of AAV allows for broad applicability of the psgMstn1 plasmid. 90 C57BL/10 male mice were divided into three groups, each with 30 mice, and two doses rAAV9-sgMstn1 and 1 × PBS were injected into male C57BL/10 mice. Three points on the thigh muscle in the left thigh in the treatment group were injected with ~ 1 × 10^10^ vg/1 × 10^11^ vg of rAAV9-sgMstn1 in 100 µL of 1 × PBS (30 µL/point), and the control group were treated with 100 µL of 1 × PBS (30 µL/point). After 6, 8, 10 weeks, 10 mice were sacrificed in each group, the expression of the tissue fluorescence were determined by a body imaging system, and the thigh muscles were collected for genomic DNA extraction, western blotting and muscle tissue sections, respectively.

### T7 endonuclease 1 (T7E1) cleavage assay and targeted deep-sequencing analysis

Genomic DNA was extracted with a Tissue and Cell Culture DNA Midi kit (TianGen, Beijing, China) according to the manufacturer’s instructions. The purified genomic DNA was used as a template to amplify a fragment of the *Mstn* gene with the following specific primers: MSTN-Test1-F: 5′-CGCCTGGAAACAGCTCCTAA-3′ and MSTN-Test1-R: 5′-TCTCATGCTTTAACACTGCCT-3′; MSTN-Test2-F: 5′-TTCTAATGCAAGCGGATGGC-3′ and MSTN-Test2-R: 5′-CACACCTACCTTTGGAGTAAGA-3′; and MSTN-Test3-F: 5′-GGATGGCAAGCCCAAATGTT-3′ and MSTN-Test3-R: 5′-ACACACCTACCTTTGGAGTAAG-3′. The fragment sizes amplified by these primer sets were 521 bp, 548 bp and 537 bp, respectively. The PCR products were digested with T7 endonuclease 1 (NEB, Boston, USA) and resolved with 1.5% agarose gel electrophoresis. The primers used for tracking of insertions and deletions (indels) by decomposition (TIDE) were the same as the MSTN-Test-F/R primers. Genomic DNA (100 ng) was used for PCR amplification with a High Fidelity 2 × PCR Master Mix (NEB). For TIDE analysis, 300 ng of PCR product was purified with a QIAquick PCR Purification Kit (Qiagen, Hilden, Mannheim, Germany) and sent for Sanger sequencing with the forward primer MSTN-Test-F. Indel values were obtained with the TIDE web tool (https://tide.deskgen.com/) as described previously. Targeted deep-sequencing analysis was performed for C2C12 cells and gDNA from mouse muscle with a PCR amplification approach. Briefly, the off-target locus was identified through the website http://www.rgenome.net/cas-offinder/. On-target or off-target locus-specific primers (Table 1) were used to amplify the editing site with Phusion High Fidelity DNA Polymerase. The resultant amplicons were separated on a 1.0% agarose gel. The bands of ~ 150 bp were extracted with a SanPrep DNA Gel Extraction kit. Then, targeted deep-sequencing of DNA products was performed by GENEWIZ Inc. In brief, the libraries were sequenced on the Illumina HiSeq platform (Illumina, Santiago, USA) in paired-end mode with a read length of 150 bp. Primary analysis was performed with built-in software, HiSeq Control Software (HCS), RTA 2.3 plus, and demultiplexing was performed with bcl2fastq 2.17. Finally, the raw data of the targeted sequencing were analyzed by bioinformatics analysts at GENEWIZ Inc. The resulting indel frequencies, sizes and distributions were then plotted with GraphPad Prism.

**Table 1:** sgRNA locus-specific primers

Primer name	Primer sequence(5′–3′)	sgRNA locus
Mus-sg1-ontarget-F	GGATGACAGCAGTGATGGCT	AAACAATCATTACCATGCCTACAGAGT
Mus-sg1-ontarget-R	ACACTAGGACAGCAGTCAGC	
Mus-sg1-1offtarget-F	TGGGAGGTCTGGGGAATGTA	ACCATGTAGACATGGTGTTGATTGTCT
Mus-sg1-1offtarget-R	AGACACCTGAGCACCCTACA	
Mus-sg1-2offtarget-F	CTCAAAAGTCCAGTGGGCCA	AAAGAACCATTTCAATGCCTAAAGAGT
Mus-sg1-2offtarget-R	TTCTGGTGAGACCCTCCCAA	
Mus-sg1-3offtarget-F	GCTCTCTGCAGGCTATGTGA	ACACAATCTTTACCATGCCCAGATGGT
Mus-sg1-3offtarget-R	CCCTGAACCATCCCACTGG	
Mus-sg1-4offtarget-F	TGCCCACTAGAATCAGCTGT	AAACAATGAATACGAAGCCTAGATAAT
Mus-sg1-4offtarget-R	TGCCACCATGAACCAAGGTA	
Mus-sg1-5offtarget-F	ACTGATATGTATGAGTGATACGCT	ATTACATGAGCTTTGTAATGATTGTTT
Mus-sg1-5offtarget-R	AGCAAGCCCTAAGAATTTTCTTTGT	
Mus-sg1-6offtarget-F	ACCTCAGCGCATCCATATGG	AAACACTCATTTCCATGCAAATACAAT
Mus-sg1-6offtarget-R	TGAAGCAGTTATGGAGTGGGT	
Mus-sg2-ontarget-F	CTCAGACCCGTCAAGACTCC	GAAAGACGGTACAAGGTATACTGGAAT
Mus-sg2-ontarget-R	GCCTGGGCTCATGTCAAGT	
Mus-sg2-1offtarget-F	GTGGGCAGGACATGTGAGAA	GCAATGCGGAACAAGGTATACAACAGT
Mus-sg2-1offtarget-R	ATGCTGTCGATTGCCTGGAA	
Mus-sg2-2offtarget-F	AGGCATTTTATAGATTCTGAGGTGA	GAAAGATGGTAAAAAGTAAACTTCAGT
Mus-sg2-2offtarget-R	TCTTCCAGATGCTCAAGGGCT	
Mus-sg2-3offtarget-F	CCTGTAGCTGATTGACTGGTAC	ACCTTTCTTTACCTAGTACTGTCTTTC
Mus-sg2-3offtarget-R	GTGGGAAGGGGCGGTAAAAA	
Mus-sg2-4offtarget-F	TGAGGGACACAGATGCCAAG	ACTGGAGTCTACATTTTACCGACTTTC
Mus-sg2-4offtarget-R	GGTATGTGTGGGGAAAACCTCTT	
Mus-sg2-5offtarget-F	ACATTAAGTCTTAACTCAGGTGCT	GAAAGACGGTACAAGGTATACTGGAAT
Mus-sg2-5offtarget-R	TCCTCTTAGCCAGCTTACAGTA	
Mus-sg3-ontarget-F	TCTCAGACCCGTCAAGACTC	GATTTGCACAAACACTGTTGTAGGAGT
Mus-sg3-ontarget-R	CCTGGGCTCATGTCAAGTTTC	
Mus-sg3-1offtarget-F	CCTGAGGTCAAACAACAAAGCT	GATTTGGAGGAACACTGTTTTGAGAGT
Mus-sg3-1offtarget-R	GGCATGAGGAGAAGGTCTCTGT	
Mus-sg3-2offtarget-F	TATTTGCTGCGTCCCTGTGG	GCTTTACACAAATACAGTTGTGAGAGT
Mus-sg3-2offtarget-R	TTAACCGCGCACCTTTTCTCC	
Mus-sg3-3offtarget-F	CAAATTGACGCTGCCAGTGT	GATTTAGACATACACTGATGTCTGAAT
Mus-sg3-3offtarget-R	GTTGTAGCGGAGGGGAGTGG	
Mus-sg3-4offtarget-F	TGTCCCCACTAATAGCTGGT	GGTGTGCACACACACAGTTGTGTGAGT
Mus-sg3-4offtarget-R	GGGGTCATGTTCTCTGGCTGA	
Mus-sg3-5offtarget-F	GTCAGACAGAGCAACTCCCG	GAACTGCACAAACCCTGGTGTGTGGGT
Mus-sg3-5offtarget-R	TCAGTGCTGGTCTCTTGCTGT	
Mus-sg3-6offtarget-F	TGACAGCAGTGCACATTGTT	GAATTGTACAAAAACTGATGTCAGAAT
Mus-sg3-6offtarget-R	AGCCCTCAAATTGCTAAGACAT	
Mus-sg3-7offtarget-F	TGAAGGTGTTGTCTGACTTCTGT	TATTTGAATAAACACTTTTGTAGGGAT
Mus-sg3-7offtarget-R	TGCTTCTTGTCTCCATGTCACTC	
Mus-sg3-8offtarget-F	AGATCCCCATCTGGTCTGTT	GATATGCACACACTCTGTTGGGGGAGT
Mus-sg3-8offtarget-R	TACGGTGTGTCCCTGTTGTGG	
Mus-sg3-9offtarget-F	CCTTCTCAGGAGAGCTGCCT	GATTTGCTCAAATACTATTTTCTGAAT
Mus-sg3-9offtarget-R	GGTGCAAGAGTCTGACTCCAT	

### Protein analysis

Protein extracts were prepared on ice by homogenization of pieces of frozen tissues or cell pellets in 500 µL of RIPA lysis buffer (50 mM Tris–HCl, pH 8.0, 150 mM NaCl, 1% Triton X-100, 1% sodium deoxycholate, 0.1% SDS and 2 mM MgCl_2_) supplemented with 1:100 protease inhibitor solution (Roche, Basel, Switzerland) by passage through a syringe. With each loose- and tight-fitting piston, the samples received 30 strokes and were then centrifuged at 1000×*g* for 5 min at 4 °C to remove debris. The supernatant (whole cell lysate) was collected. For the isolation of membrane fractions, the supernatant was further centrifuged at 13,200×*g* for 20 min at 4 °C, and the pellet was resuspended (2 µL/mg tissue) in sample buffer (2.7 M urea, 3.3% SDS and 0.167 M Tris, pH 6.7). The protein concentration was estimated with a BCA assay. For protein detection, 30 µg of sample was separated with 10% SDS-PAGE and then transferred to a polyvinylidene fluoride membrane. After incubation in 5% nonfat milk for 1 h, the membrane was incubated with rabbit polyclonal anti-MSTN/GAPDH antibody (1:1000, Bioss Antibodies, China, catalogue number: bs-23012R/bs-2188R) overnight at 4 °C. Then membranes were incubated with horseradish peroxidase-conjugated goat anti-rabbit (1:2000, Bioss Antibodies, catalogue number: bs-0295G) antibodies for 1 h at room temperature. The target proteins were detected with Luminata™ Crescendo immunoblotting HRP Substrate (Millipore, USA).

### Histology analyses

The quadriceps and adductors muscles were fixed in para-formaldehyde and embedded in paraffin for further histopathological investigations. Formalin-fixed quadriceps and adductors muscles sections were stained to investigate the density and diameter of muscle fibers by hematoxylin and eosin according to a standard protocol (Feldman and Wolfe 2014). After the staining procedure, the slides were scanned with a microscope. At least five fields at 40 × /200 × magnification were definite selected from each section and ten sections in each group for imaging. The muscle mass at the same muscle position was observed in different groups of mice. The cross-sectional area of each quadriceps or adductors muscle in each section was analyzed, and then the average cross-sectional area (mm2) of individual muscle cell was calculated. All data were obtained and analyzed with Image Pro Plus 6.0 software.

### Muscle weights of mice

The experimental mice were weighed weekly from the first week to the eighth week. The quadriceps and adductor muscles from the left thigh in the experimental mice were dissected at 10 weeks. The quadriceps and adductors muscles were weighed on electronic scales and the weight was recorded for each muscle. Ten mice were weighed per group.

### Statistical analyses

Data are expressed as the mean ± standard error of the mean. Unpaired Student’s *t* test and one-way ANOVA were used for group comparisons with Prism 6 (GraphPad). *P values less than 0.05 and greater than 0.01 were considered significant. **P value less than 0.01 was considered to be of greater significance.

## Results

### Construction of new CRISPR/SaCas9 vectors and location of *Mstn* knockout targets

In our experiment, we focused on the design of a compact CRISPR/SaCas9 vector, pX601-EF1α:SaCas9-eGFP-tRNA:sgRNA plasmid suitable for AAV delivery. The pX601-EF1α:SaCas9-eGFP-tRNA:sgRNA plasmid contains an EF1α promoter controlling expression of the SaCas9 coding region fused to a self-cleaving P2A sequence attached to an eGFP sequence, and it contains a constitutively expressing sgRNA expression cassette controlled by a tRNA promoter. The P2A sequence enables the SaCas9 and the eGFP domain to yield two independent proteins from the same cistron, and the translated proteins from the two genes retain their functions. This approach can be used to indirectly understand the transfection ability of recombinant AAV and the gene editing effect mediated by SaCas9 protein through the expression level of GFP protein (Fig. 1a).

The sgRNAs were designed to target the mouse *Mstn* locus (sgMstn1/sgMstn2/sgMstn3) (Fig. 1b). We chose to target the *Mstn* locus because it is a well-studied gene known for its important role in muscle of overgrowth and is important in research on some muscular atrophic diseases. The three sgRNAs are located at the N-terminal of the first and second exon of *Mstn* gene, and these sites play an important role in the synthesis of MSTN precursor proteins.

### The reconstructed CRISPR/SaCas9 vector exhibits efficient genome editing in vitro

**Fig. 2:**
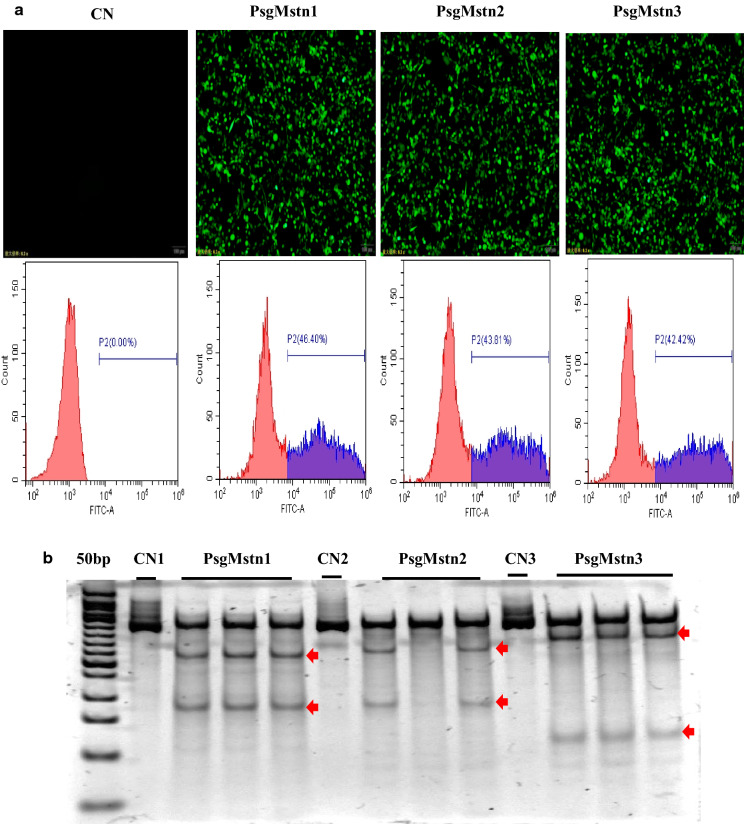

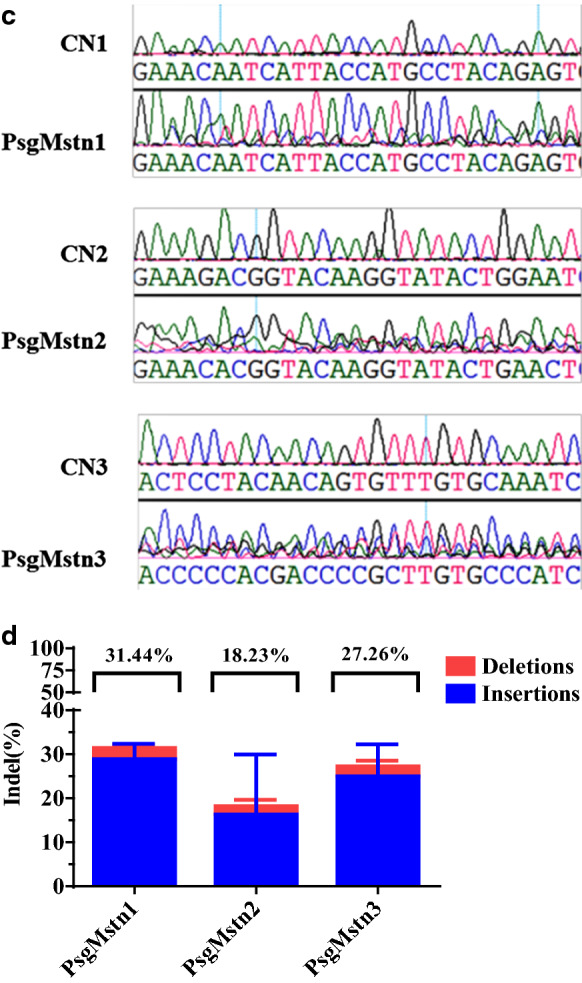
The SaCas9/CRISPR system exhibits efficient genome editing in vitro. **a** pX601-EF1α:SaCas9-eGFP-tRNA:sgRNA plasmid was transfected into NIH/3T3 cells. For the treatment group (PsgMstn1, PsgMstn2, PsgMstn3), green fluorescence were observed to determine eGFP expression. No green fluorescence was detected in the control group (CN). The eGFP fluorescence expression of transfected cells were calculated by flow cytometry. The abscissa represents the value of the relative strength of the eGFP fluorescence signal, the ordinate is the number of cells, and P2 shows the percentage of eGFP fluorescent cells relative to the total number of cells. **b** The NIH/3T3 cells of experimental groups were harvested, and genomic DNA were isolated and purified. The 521 bp, 548 bp and 537 bp regions of the sgMstn1/sgMstn2/sgMstn3 locus, including the site targeted for editing, were PCR amplified from genomic DNA and analyzed for genome editing with a T7 endonuclease digestion identification method. The top band on the gel is uncut DNA at 521 bp, 548 bp and 537 bp in length, and the two smaller bands were edited DNA at 181 and 340 bp in length for sgMstn1, 185 and 352 bp in length for sgMstn2, and 122 and 415 bp in length for sgMstn3 (red arrow indicated). The nucleic acid marker was a 50 bp DNA ladder. **c** The sgMstn1/sgMstn2/sgMstn3 knockout sequence peak was obtained by Sanger sequencing. For a control, genomic DNA of the wild type NIH/3T3 cells was sequenced. **d** Stacked histogram showing the percentage distribution of indels at sgMstn1, sgMstn2 and sgMstn3 in the treatment groups, as measured by sequencing analyses. Data represent means ± SD from three technical replicates

To examine the genome editing capabilities of the reconstructed CRISPR/SaCas9 vector, we transfected NIH/3T3 cells with the plasmid psgMstn1/psgMstn2/psgMstn3 in the treatment groups. The expression of eGFP from the transfected plasmid psgMstn1/psgMstn2/psgMstn3 was confirmed by cellular immunofluorescence in the NIH/3T3 cells. The control cells were wild type cells cultured in the same batch. A flow cytometry experiment was performed on NIH/3T3 cells transfected with the psgMstn1/psgMstn2/psgMstn3 plasmids. The expression efficiency of psgMstn1/psgMstn2/psgMstn3 plasmids was 46.40%, 43.81% or 42.42%, respectively (Fig. 2a). The results confirmed that the recombinant vectors expressed effectively. Genomic DNA was isolated, and the 521 bp, 548 bp and 537 bp regions from the sgMstn1/sgMstn2/sgMstn3 loci were PCR amplified and examined for SaCas9-mediated editing with a T7 endonuclease digestion identification method. With this assay, if no editing occurred, a single band was visible on the gel. The results were as shown in the control groups. When editing did occur, the DNA was cut at the location of the editing, thus resulting in two smaller bands of 181 and 340 bp in length for sgMstn1; 185 and 352 bp in length for sgMstn2; and 122 and 415 bp in length for sgMstn3 (Fig. 2b). For TIDE analysis, PCR products were sent for Sanger sequencing with the forward primer MSTN-Test1/2/3-F. The sequencing results of psgMstn1/psgMstn2/psgMstn3 plasmids showed that compared with the ordered, single peaks of normal cell gene, the knockout cell gene had chaotic peaks due to random mutations at the knockout site. (Fig. 2c). Indel values were obtained with the TIDE web tool (https://tide.deskgen.com/). The control group did not exhibit editing of the *Mstn* locus. However, cells transfected with psgMstn1/psgMstn2/psgMstn3 plasmids displayed an average of 31.44%, 18.23% or 27.26% editing, respectively (Fig. 2d). Genome editing percentages were averaged from three independent samples per group. From the above data, we determined that all three plasmids had gene editing ability. Compared with that of sgMstn2 and sgMstn3, the gene editing efficiency of sgMstn1 was higher, and the knock-off efficiency was lower. Therefore, sgMstn1 was selected as the first choice for in vivo and in vitro targeting of packaged rAAV vectors.

### The rAAV-DJ/8-SaCas9 efficiently expresses the eGFP fluorescence in vitro

**Fig. 3:**
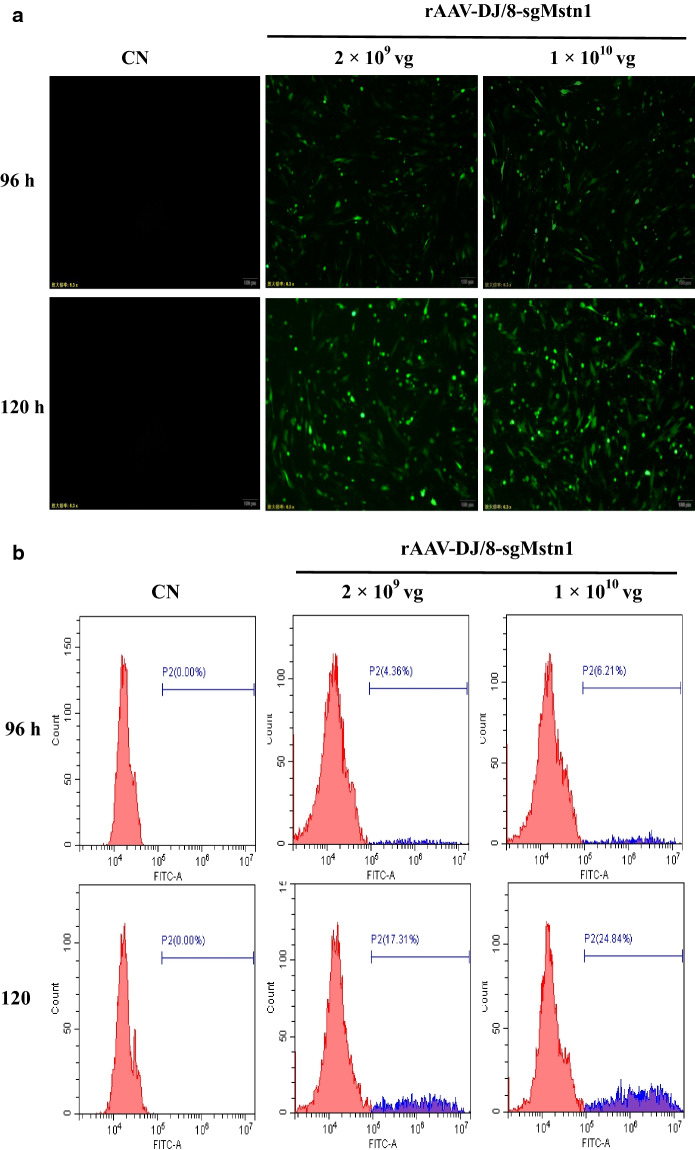
The rAAV-DJ/8-SaCas9 efficiently expresses the eGFP fluorescence in vitro. **a** The treatment groups infected into C2C12 cells with ~ 2 × 10^9^ vg/1 × 10^10^ vg rAAV-DJ/8-sgMstn1 and the control group was wild type cells cultured in the same batch. At 96 h, 120 h later, green fluorescence were observed to determine eGFP expression. **b** The eGFP fluorescence expression of infected cells were calculated by flow cytometry. The abscissa represented the value of the relative strength of the eGFP fluorescence signal, the ordinate was the number of cells, and P2 showed the percentage of eGFP fluorescent cells relative to the total number of cells

We infected C2C12 cells with ~ 2 × 10^9^ vg/1 × 10^10^ vg of rAAV-DJ/8-sgMstn1 in the treatment groups. The control group comprised wild type C2C12 cells cultured in the same batch. All cells were observed and harvested 96 h, 120 h later. The expression of eGFP in the treatment groups were confirmed by cellular immunofluorescence in C2C12 cells. No eGFP expression was observed in the control group (Fig. 3a). A flow cytometry experiment was performed on C2C12 cells infected with the rAAV-DJ/8-sgMstn1. The eGFP expression efficiency of the treatment groups with 2 × 10^9^ vg rAAV-DJ/8-sgMstn1 was 4.36%, 17.31% at 96 h, 120 h later and 1 × 10^10^ vg rAAV-DJ/8-sgMstn1 was 6.21%, 24.84% at 96 h,120 h later, respectively. No eGFP expression was observed in the control group (Fig. 3b). The eGFP expression efficiency of the treatment groups were prominent in vitro.

### The rAAV-DJ/8-SaCas9 efficiently targets the *Mstn* site in C2C12 cells

**Fig. 4:**
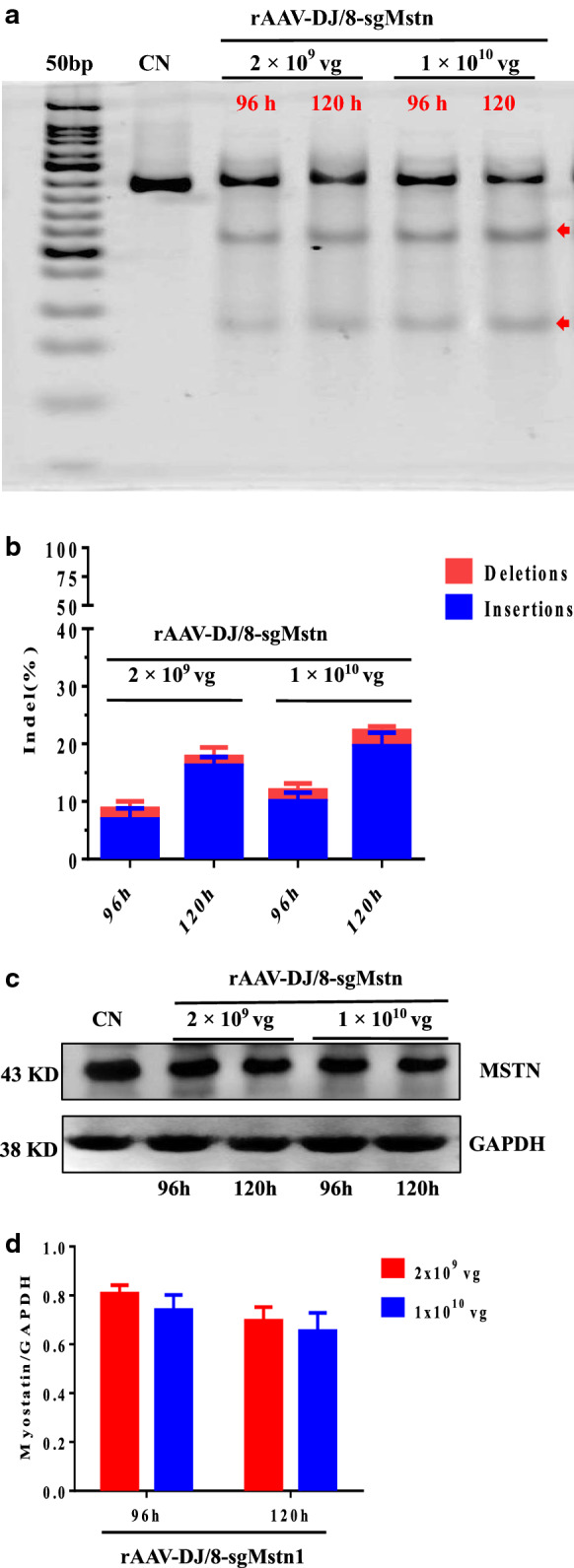
The rAAV-DJ/8-SaCas9 system efficiently targets the *Mstn* site in C2C12 cells. **a** The treatment groups infected into C2C12 cells with two dose rAAV-DJ/8-sgMstn1 and the control group was wild type cells cultured in the same batch. A 521 bp region of the sgMstn1 locus including the site targeted for editing was PCR amplified from the genomic DNA and analyzed for genome editing with a T7 endonuclease digestion identification method. The top band on the gel was uncut DNA at 521 bp in length, and the two smaller bands were edited DNA at 181 and 340 bp in length for sgMstn1 at 96 h, 120 h post infection (red arrow indicated). The nucleic acid marker was a 50 bp DNA ladder. **b** The percentage distribution of indels at sgMstn1 in C2C12 mutant cells, as measured by sequencing analyses. Data represent means ± SD from three technical replicates. **c** The treatment groups and the control group were analyzed by western blotting with an anti-MSTN antibody. Anti-GAPDH was used as a loading control. **d** Stacked histogram showing the percentage distribution of MSTN expression in different groups. The MSTN value of each group was divided by the corresponding GAPDH value, and then the data for each group were divided by the same multiple to normalize by the control group value. Data represent means ± SD from three technical replicates

We have demonstrated that *Mstn* site knockout occurs at the cellular and molecular level in C2C12 cells. Genomic DNA was isolated from a 521 bp region from the sgMstn1 locus, PCR amplified and examined for SaCas9-mediated editing with a T7 endonuclease digestion identification method. The results indicated two smaller bands 181 and 340 bp in length for sgMstn1 (Fig. 4a). For TIDE analysis, the 2 × 10^9^ vg of rAAV-DJ/8-sgMstn1 resulted in 8.72%, 17.75% editing at 96 h, 120 h, and the 1 × 10^10^ vg of rAAV-DJ/8-sgMstn1 resulted in 11.94%, 22.22% editing at 96 h, 120 h. The control group did not exhibit editing of the *Mstn* locus (Fig. 4b). Genome editing percentages were averaged from three independent samples per group. Compared with the control group, the treatment groups infected with rAAV-DJ/8-sgMstn1 showed decrease in MSTN protein levels (Fig. 4c). A stacked histogram showing the percentage distribution of MSTN expression in two dose treatment groups indicated 80.67%, 74% at 96 h, and 69.67%, 65.33% at 120 h (Fig. 4d). Data represent the means ± SD from three technical replicates. Efficient editing and MSTN protein reduction indicated the success of the delivery and activity of rAAV-DJ/8-sgMstn1 at the *Mstn* locus, and demonstrated that gene editing of the cells were effective by the rAAV-DJ/8-sgMstn1.

### The rAAV9-SaCas9 efficiently expresses the eGFP fluorescence in vivo

**Fig. 5:**
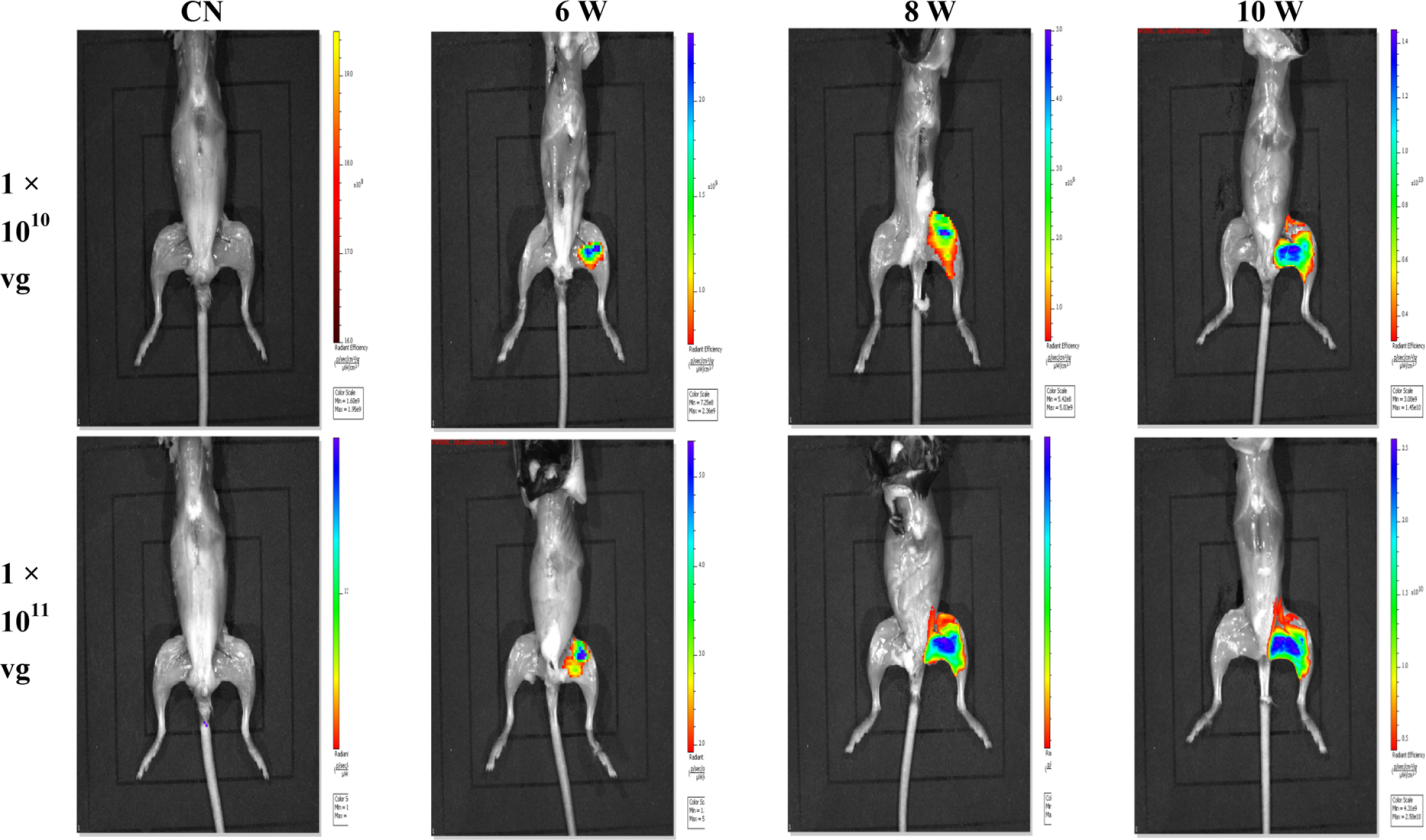
The rAAV9-SaCas9 efficiently expresses the eGFP fluorescence in vivo. For two dose rAAV9-SaCas9, the expression range was 2 × 10^10^–5 × 10^10^photons/second/cm^2^. The images were shown at 6, 8, 10 weeks post-injection (dpi). Owing to the weak fluorescence expression of eGFP, fluorescence imaging results were not visible through the mouse fur. Therefore, we killed the mice first and then stripped off the fur for fluorescence imaging. To exclude negative expression in the thigh, the first image was a control group placed in front of the rAAV9-sgMstn1 treatment groups (ventral position)

We sought to assess whether our SaCas9 system could be used for steady expression in vivo. In these experiments, we produced rAAV9-sgMstn1, pseudotyped as 9 serotype. In the two treatment groups, rAAV9-sgMstn1 were infected into the left thigh muscle in C57BL/10 male mice, each at a titer of ~ 1 × 10^10^ vg/1 × 10^11^ vg. We found that eGFP fluorescence expression was detected 6 weeks later in the treatment groups, and the expression of eGFP was more obvious with the increase of time. In addition, the fluorescence expression of eGFP was higher in the high-dose group at the same time point, and no fluorescence expression was detected in the control group (Fig. 5).

### The rAAV9-SaCas9 efficiently targets the *Mstn* site in mice thigh muscle

**Fig. 6:**
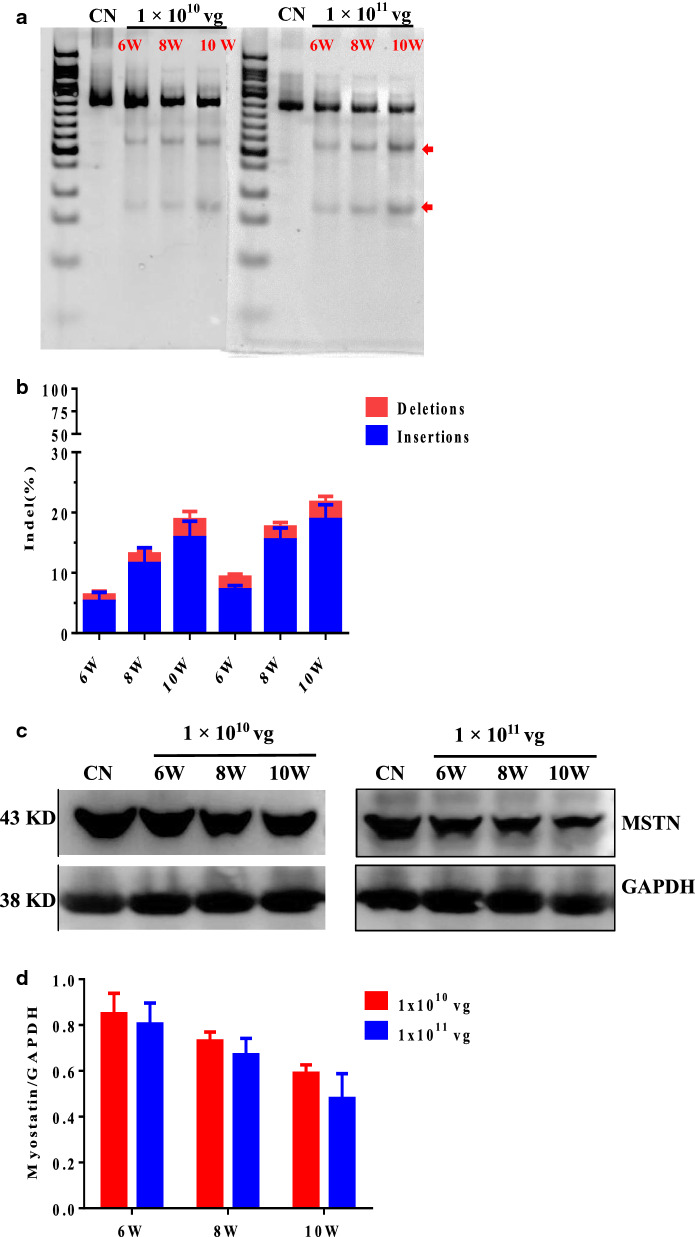
The rAAV9-SaCas9 system efficiently targets the *Mstn* site in C57BL/10 male mice thigh muscle. **a** A 521 bp region of the sgMstn1 locus including the site targeted for editing was PCR amplified from the genomic DNA and analyzed for genome editing with a T7 endonuclease digestion identification method. The top band on the gel was uncut DNA at 521 bp in length in all groups, but the two smaller bands were edited DNA at 181 and 340 bp in length for sgMstn1 in the treatment groups (red arrow indicated). The nucleic acid marker was a 50 bp DNA ladder. **b** The percentage distribution of indels at sgMstn1 in thigh muscle in mice with different dose rAAV9-sgMstn1, as measured by sequencing analyses. Data represent means ± SD from three technical replicates. **c** Mice left thigh muscles were treated with ~ 1 × 10^10^ vg/1 × 10^11^ vg of the rAAV9-sgMstn1 and 1and, analyzed by western blotting with anti-MSTN antibody at 6, 8, 10 weeks post infection. Anti-GAPDH was used as a loading control. **d** Stacked histogram showing the percentage distribution of MSTN expression in mice thigh muscles treated with different dose rAAV9-sgMstn1. The MSTN value of each group was divided by the corresponding GAPDH value, and then the data of each group were divided by the same multiple to normalize by the control group value. Data represent means ± SD from three technical replicates

We have demonstrated that *Mstn* site knockout occurs at the cellular and molecular level in mice thigh muscle. Genomic DNA from mice muscle was isolated, and a 521 bp region from the sgMstn1 locus was PCR amplified and examined for SaCas9-mediated editing with a T7 endonuclease digestion identification method. The results revealed two smaller bands 181 and 340 bp in length for sgMstn1 in the treatment groups, which were absent in the control group (Fig. 6a). For TIDE analysis, the 1 × 10^10^ vg of rAAV9-sgMstn1 displayed 6.31%, 13.11% and 18.8% editing at 6, 8 and 10 weeks, and the 1 × 10^11^ vg of rAAV9-sgMstn1 displayed 9.29%, 17.59% and 21.68% editing at 6, 8 and 10 weeks (Fig. 6b). Muscle from mice that received the 1 × PBS did not exhibit editing of the *Mstn* locus. The genome editing percentages were averaged from three independent samples per group. Compared with the control group muscles, MSTN protein levels were reduced in the muscle infected with ~ 1 × 10^10^ vg/1 × 10^11^ vg of the rAAV9-sgMstn1 (Fig. 6c). A stacked histogram showing the percentage distribution of MSTN expression in muscle after infection with 1 × 10^10^ vg of the rAAV9-sgMstn1 indicated 85%, 73.2%, 59.8% at 6, 8, 10 weeks. And the percentage distribution of MSTN expression in muscle after infection with 1 × 10^11^ vg of the rAAV9-sgMstn1 indicated 80.43%, 67.5%, 51.33% at 6, 8, 10 weeks (Fig. 6d). The data represent means ± SD from three technical replicates. Efficient editing and MSTN protein reduction indicated the success of the delivery and activity of rAAV9-sgMstn1 at the *Mstn* locus in mice. Moreover, the editing effect of the two treatment groups was more obvious with the increase of time, while that of the high-dose group was relatively higher.

### The rAAV9-SaCas9 effectively edited genes and improved muscle mass in mice

**Fig. 7:**
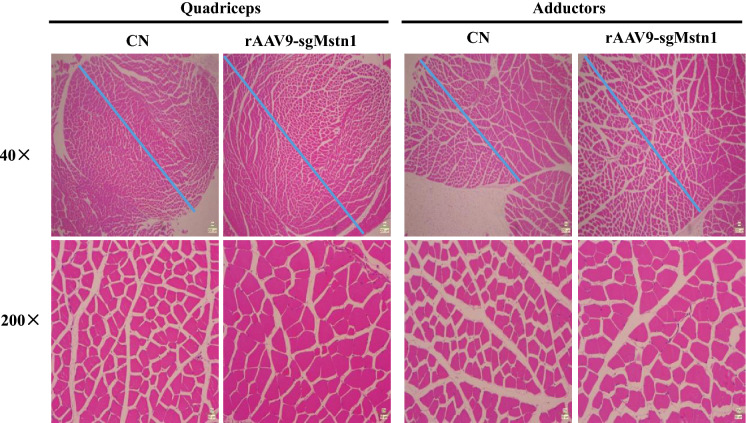
The rAAV9-SaCas9 efficiently improves muscle mass in mice. Sections were cut from each quadriceps and adductors muscle and stained with hematoxylin and eosin (H & E) to visually determine the myofiber shape. Representative images of H & E staining of CN and rAAV9-sgMstn1 treated muscle sections are shown. The cross-sectional area of the muscle changed significantly in different groups, and the blue line showed the span of the same area (scale bar = 200 μm). The area of individual cells also changed greatly in different groups (scale bar = 50 μm)

Hematoxylin and eosin staining of the 1 × 10^11^ vg rAAV9-sgMstn1 treatment group and the control group were performed, and the transverse sections of the quadriceps and adductors muscles showed different density distributions. The total muscle mass of the treatment group had significantly enlarged than the control group (proportional scale: 200 µm). And the cross-sectional area of each muscle fiber of the treatment group were substantially added (proportional scale: 50 µm) (Fig. 7).

### *Mstn* gene was knocked out by the rAAV9-SaCas9 in thigh muscles to increase muscle mass

**Fig. 8:**
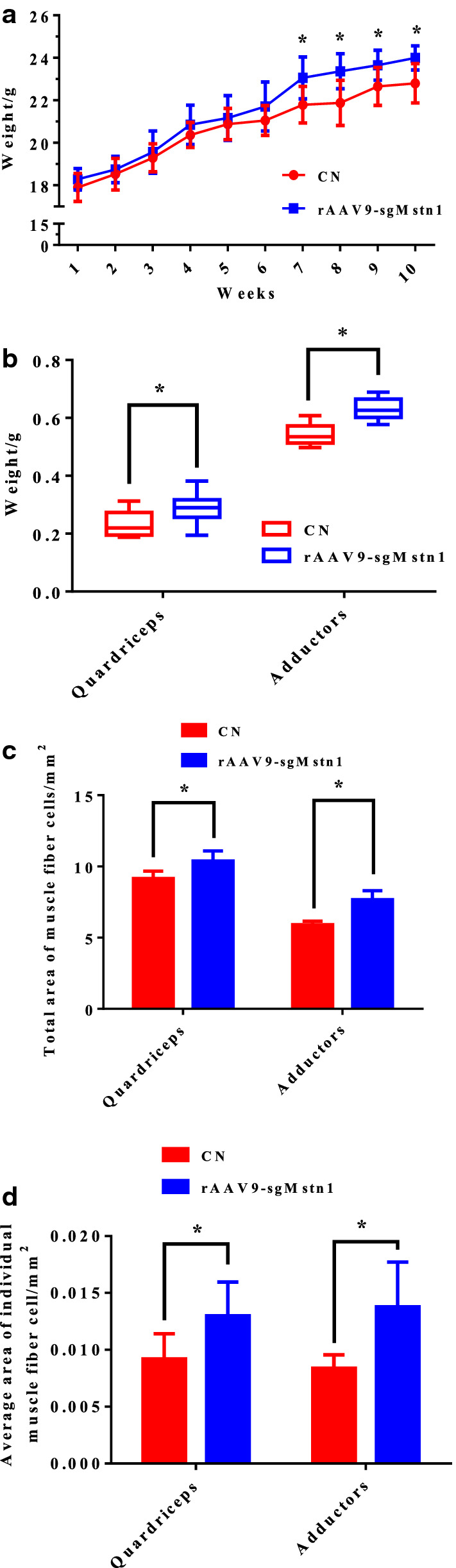
The rAAV9-SaCas9 changed the size and number of muscle cells. **a** Changes in body weight from 1 to 10 weeks in the rAAV9-sgMstn1 treated group and the control group. Compared with the control group, the body weight in the rAAV9-sgMstn1 treated group was significantly increased. N = 10/group, *0.01 < P < 0.05, **P < 0.01 for the rAAV9-sgMstn1-treated group compared with the control group. **b** The weights of the quadriceps and adductor muscle in the different groups at 10 weeks. N = 10/group, *0.01 < P < 0.05, **P < 0.01 for the rAAV9-sgMstn1-treated group compared with the control group. **c** and **d** The total area of muscle fiber cells (mm^2^) and average area of individual muscle cell (mm^2^) were measured. N = 10/group, *0.01 < P < 0.05, **P < 0.01 for the rAAV9-sgMstn1 treated group compared with the control group

We detected the changes of body weight in C57BL/10 mice after *Mstn* was knocked out. Compared with the control group, the body weight of the 1 × 10^11^ vg rAAV9-sgMstn1 treatment group were substantially added, especially in the 7–10 weeks (Fig. 8a). In addition, we clearly found the weight change of quadriceps and adductor. Compared with the control group, treatment with 1 × 10^11^ vg of the rAAV9-sgMstn1 significantly increased the quadriceps weight (0.01 < P < 0.05) and adductors weight (0.01 < P < 0.05) at 10 weeks (Fig. 8b). These results revealed muscle weight increase in the *Mstn* knockout muscle. According to analysis of the quadriceps and adductors muscle sections, we observed that the total area of quadriceps and adductors muscles in the treated group were significantly greater than those in the control group (0.01 < P < 0.05) at 10 weeks (Fig. 8c). And the average area of individual muscle fiber cell in the treated group was significantly greater than it in the control group (0.01 < P < 0.05) at 10 weeks (Fig. 8d). The results revealed that the area of muscle fibers increased, and muscles enlarged, with decreased MSTN expression in muscle tissues.

### SaCas9 is highly specific in vivo

**Fig. 9:**
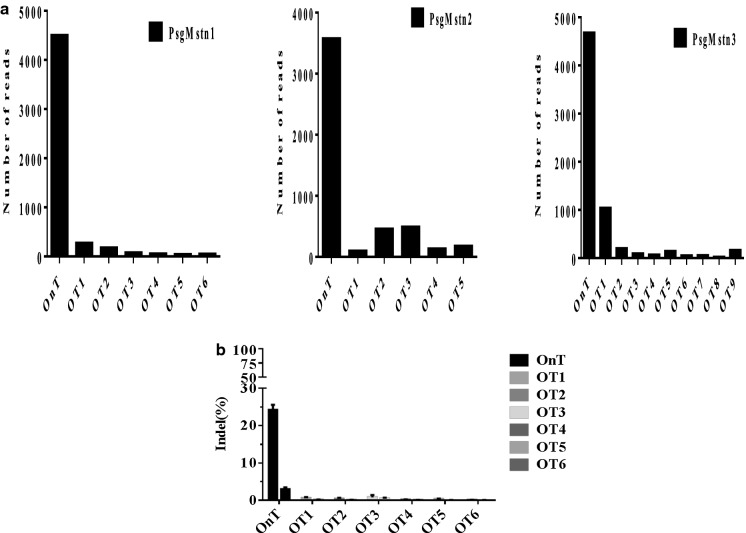
SaCas9 is highly specific in vivo. **a** Numbers of sequencing reads for the on-target (OnT) and off-target (OT) sites of sgMstn1, sgMstn2 and sgMstn3 in C2C12 cells. **b** Targeted deep-sequencing to measure the muscle tissues at each OT site of sgMstn1

The possibility of off-target edits is a major concern in therapeutic CRISPR/Cas9 genome editing. Some researchers have found that SaCas9 is a naturally highly occurring genome editing platform in mice (Xu et al. 2019; Ohmori et al; 2017). Using targeted deep-sequencing of mouse cell DNA products obtained by GENEWIZ Inc., we screened for off-target sites in the mouse genome to determine whether SaCas9 maintained its minimal off-targeting profile in mice cells. NIH/3T3 cells were transfected with psgMstn1, psgMstn2 and psgMstn3 plasmids. The resulting genomic DNA was subjected to sequencing analysis. Sequencing revealed few off-target sites in the mouse genome, a result consistent with our previous observations in mouse cells. Six potential off-target sites were identified for sgMstn1, five for sgMstn2 and another nine for sgMstn3, with a low probability of off-target sites in the mouse genome (Fig. 9a). We concluded that SaCas9 editing is intrinsically specific. We also performed targeted deep-sequencing with genomic DNA from the muscles of mice to validate these off-target sites. According to this more sensitive readout, indels detected more accurately above background at all of the off-target locus 1 sites with *Mstn* (Fig. 9b). These results indicated that rAAV9-sgMstn1 is a promising and safe candidate for in vivo applications.

## Discussion

Several CRISPR/Cas9-based-inducible systems have been developed, but few have been developed for the SaCas9 variant. Since the characterization of SaCas9 (Ran et al. 2015), several groups have extended its application in targeted mutagenesis in a variety of models such as plants, mice and zebrafish (Kaya et al. 2017; Xu et al. 2018; Feng et al.^2016^). Because of its smaller size, SaCas9 provides substantial advantages in the delivery and expression of Cas9, especially when AAVs are used. Recent work has shown that SaCas9 has higher activity than the other Cas9 variants such as SpCas9 and FnCpf1 (Xie et al. 2018).

To our knowledge, there are currently few available single AAV viruses containing both the Cas9 and sgRNA. Among them, the rAAV-SaCas9 system has been shown to be effective for gene editing in vivo (Ran et al. 2015). These systems not only provide comparable editing levels to those of other SpCas9 systems, but also have the advantage of being within the AAV packaging limit. Moreover, researchers can manipulate gene expression at precise sites and to precise degrees of expression. Therefore, these SaCas9 systems can probably be used to edit genes at specific sites to improve tissue traits and treat chronic diseases (De Caneva et al. 2019; Xu et al. 2019). First, we report the development of an SaCas9 system with a different sgRNA promoter (tRNA pro ~ 72 bp) and SaCas9 promoter (EF1α pro ~ 212 bp) that can be delivered to cells via rAAV. This single vector system contains a smaller Cas9, a fluorescent protein and a gRNA expression cassette. Only one virus is needed to mediate genome editing in animals or cell lines. We believe that this system should be extremely useful and may be used separately or in combination with other expression cassettes in experiments.

We observed favorable gene editing efficiency by AAV in vitro across our experiments. Through infection of C2C12 cells, we observed fluorescence expression of the eGFP protein in rAAV-DJ/8-sgMstn1. One study has indicated that when the SaCas9 system targets the HT1080 cell line in vitro, the genome editing rate is ~ 40–50% (Morsy et al. 2017). In contrast, our system showed relatively low editing efficiency ~ 17.31% in *vitro* with the same amount of virus, but by increasing the amount of virus, we substantially improved the editing efficiency approximately ~ 24.84%. So we found that increasing the amount of rAAV-DJ/8-sgMstn1 improved the expression of eGFP protein and the efficiency of SaCas9 protein editing. We also found that rAAV-SaCas9 was expressed stably in cells for a long time in our experiments. Therefore, genome editing efficiency can be improved by increasing the expression time and titer of rAAV-DJ/8-sgMstn1.

Because of differences in AAV serotypes, transcription factors and injection sites, AAV-mediated delivery of transgenes in tissues often differs (Huang et al. 2017). In addition, the titer of the AAV virus and the method of virus injection also greatly affect the efficiency of AAV-mediated transgene delivery. Intramuscular injection was used because it has a highly targeted effect on tissues. The effect is more easily analyzed by comparing the differences in muscle tissues, cells and corresponding proteins without considering the overall effect on local areas. In addition, systemic injection may cause adverse effects specific to nerves, immune cells, hormones and other factors, and may cause a large area of off-target editing and even cancer mutations in offspring. *Mstn* knockout in mice also resulted in changes in body characteristics. Therefore, we focused on local targeted knockout experiments in gene editing. In our experiments, we infused our rAAV-SaCas9 system into the left thigh in C57BL/10 male mice. Therefore, genome editing was restricted to the SaCas9 system within this region. We found that the higher the rAAV9-sgMstn1 titer, the higher the fluorescence intensity of eGFP protein. Moreover, we were able to clearly observed the location of the rAAV9-sgMstn1 through eGFP protein fluorescence. Although eGFP fluorescence of the rAAV was detected, the fluorescence intensity was not strong and it was easy to be interfered by the fur. Therefore, the expression of this eGFP fluorescence needs to be improved.

We observed the feasibility and effectiveness of genome editing in these systems, which achieved average ~ 20.24% genome editing. These editing efficiencies are higher than those in previous studies from laboratory examining SaCas9 genome editing (Wei et al. 2016; Kumar et al. 2018). In agreement with the results of in vitro experiments, increasing the amount of recombinant rAAV in vivo also promoted the genome editing efficiency of the rAAV. The genome editing efficiency in the high virus titer group was 17.59% at 8 weeks, and the greatest amount of genomic editing was ~ 21.68%. Therefore, within a certain period of time, when the amount and expression time of the rAAV increased, the gene editing in vivo achieved the best effect.

Muscle wasting occurs with aging and in a wide range of catabolic diseases, such as cancer, diabetes, chronic renal disease and heart failure, which can dramatically decrease quality of life and increase disease mortality (Wei et al. 2016). Recently, in vivo genome editing targeting muscle tissues with CRISPR-Cas systems has shown efficacy in a mouse model of Duchenne muscular dystrophy (Lim et al. 2018). The researchers found that by blocking the MSTN protein expression and myostatin pathway of some skeletal muscle cells, these cells should be able to prevent the atrophy of target cells and adjacent cells, thus maintaining muscle function to a certain extent (Cohen et al. 2015). Our constructed rAAV-SaCas9 system has been shown to be effective for *Mstn* editing of thigh muscles. Moreover, the rAAV-SaCas9 system significantly improved muscle cells by knocking out the *Mstn* gene. This laid a foundation for the clinical application of the rAAV-SaCas9 system. Thomas and Joulia et al. have found that MSTN up-regulates the activity and level of cyclin-dependent kinase inhibitors, thus preventing G1 phase to S phase transition, inhibiting the proliferation of muscle cells, and decreasing the number of muscle fibers (Thomas et al. 2000; Joulia et al. 2003). McPherron and coworkers have found that mice with gene knockout of *Mstn* have significantly enlarged muscles. In the mutant mice, the number of muscle fibers was 86% greater than that in wild-type mice, thus indicating that *Mstn* knockout causes muscle fiber growth and hypertrophy (McPherron et al. 1997). The results of our experiment are consistent with that conclusion. Treatment with rAAV9-Mstn1 resulted in significantly higher body weight, quadriceps weight and adductor weight than those in the normal group. Through analysis of muscle tissue, we confirmed that knockout the *Mstn* gene changed the volume and number of muscle fibers, an effect particularly important for improving muscle mass.

In summary, gene editing for all of the systems could probably be optimized further by using highly efficient sgRNAs and high titer rAAV, increasing the incubation time of these viruses in vivo, and using serotypes of AAV that transduce target cells with high efficiency. In this paper, we demonstrate that the constructed rAAV-SaCas9 system confer stable expression in vitro and in vivo. Moreover, The fluorescence intensity of eGFP protein and the gene editing efficiency of SaCas9 protein increased with increasing rAAV-SaCas9 titer. The fluorescence intensity of the eGFP protein was also used to determine the infection effects and the location of the rAAV-SaCas9. In addition, we edited mice muscle with a high dose of rAAV9-SaCas9 virus, which effectively improved the muscle properties of mice. The results show that the rAAV-SaCas9 system has great potential in disease treatment and genetic modification.
